# Fragility of Bone Material Controlled by Internal Interfaces

**DOI:** 10.1007/s00223-015-9978-4

**Published:** 2015-03-14

**Authors:** Wolfgang Wagermaier, Klaus Klaushofer, Peter Fratzl

**Affiliations:** 1 Department of Biomaterials, Max Planck Institute of Colloids and Interfaces, Research Campus Golm, 14424 Potsdam, Germany; 2 First Medical Department, Hanusch Hospital, Ludwig Boltzmann Institute of Osteology at Hanusch Hospital of WGKK and AUVA Trauma Centre Meidling, Heinrich Collin Str. 30, 1140 Vienna, Austria

**Keywords:** Bone fragility, Collagen, Bone mineral, Osteoporosis, Bone material quality

## Abstract

Bone material is built in a complex multiscale arrangement of mineralized collagen fibrils containing water, proteoglycans and some noncollagenous proteins. This organization is not static as bone is constantly remodeled and thus able to repair damaged tissue and adapt to the loading situation. In preventing fractures, the most important mechanical property is toughness, which is the ability to absorb impact energy without reaching complete failure. There is no simple explanation for the origin of the toughness of bone material, and this property depends in a complex way on the internal architecture of the material on all scales from nanometers to millimeters. Hence, fragility may have different mechanical origins, depending on which toughening mechanism is not working properly. This article reviews the toughening mechanisms described for bone material and attempts to put them in a clinical context, with the hope that future analysis of bone fragility may be guided by this collection of possible mechanistic origins.

## Introduction

Bone fragility is a serious condition that may be due to genetic disorders, such as osteogenesis imperfecta [1, 2], or metabolic diseases, such as osteoporosis [3]. A very important contribution to preventing fragility and fracture is made by the bone mass and geometry. However, the properties of the mineralized matrix also play an important role. It is quite impressive to see the mechanical quality of bone under normal conditions, given that it essentially consists of a brittle mineral (carbonated apatite), a polymer (collagen type I) and water. The reason is mainly the complex three-dimensional architecture of the bone material, which offers many toughening mechanisms that reduce the fragility of the components. This means that the toughness of the bone material depends essentially on the way in which mineralized collagen fibrils are assembled into building blocks with many length scales, as well as the quality of the interfaces between these building blocks. Conversely, this implies that increased fragility does not necessarily result from a deterioration of the average (bulk) properties of the material but may result from a modification of this assembly and, in particular, of the interfaces at all scales, e.g., between collagen and mineral, between collagen fibrils, between lamellae, between osteons, etc. Given that these internal interfaces cover only a tiny fraction of the material volume, searching for the origin of fragility may sometimes seem like searching for a needle in a haystack.

**Fig. 1:**
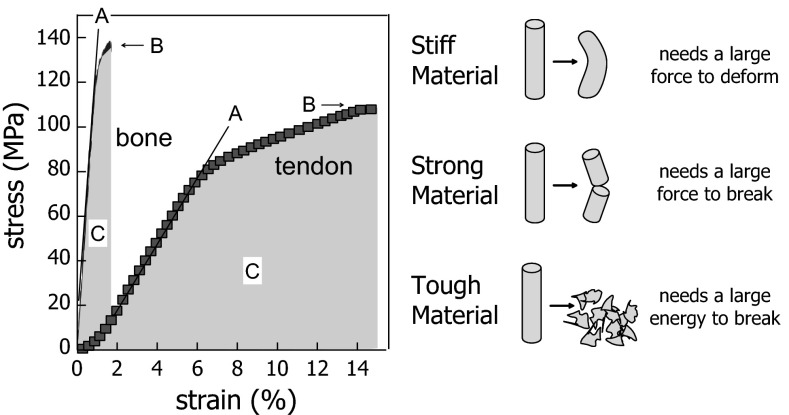
Typical stress-strain curve of bone material and tendon (*left*) and definitions of stiffness, strength and toughness (*right*). Stiffness is the resistance against (small) deformation and corresponds to the slope (**a**). Material stiffness is often measured by the elastic modulus (or Young’s modulus). Strength is the maximum stress the material can sustain before failure (**b**), and a rough measure for toughness is the energy to failure [*shaded area under the curve* (**c**)]. Clearly, bone is stiffer but tendon is tougher (*left panel*). Figure adapted and reprinted from [7] with permission from Springer Science and Business Media

Bone material normally combines sufficient stiffness and strength with high toughness (see Fig. 1 for definitions), all of which are needed to withstand the low-energy trauma likely occurring in the daily life. In particular, stiffness is needed to prevent bending of our bones under the load of the body and toughness to absorb as much energy as possible during impacts, thus retarding bone fracture. Unfortunately, these properties are contradictory [4]. High stiffness and strength are often accompanied by low toughness (as in brittle ceramics), while tough materials are usually deformable and thus cannot be very stiff. Mineral (which has properties close to ceramics) provides stiffness to bone (see Fig. 1), while the organic matrix reduces the inherent brittleness of the mineral [5]. When it comes to the description of fragility, other factors also need to be taken into account. As already mentioned, fracture resistance depends on material structures and their interfaces at all scales. This means that, although a number of toughening mechanisms have been identified [6], a complete theoretical description of fracture resistance does not exist. The purpose of this review is to define a few key concepts about the relation between bone material structure and fragility and to summarize the most important toughening mechanisms described in recent studies. Most of these mechanisms involve interfaces between bone substructures; therefore, these structural features need to be primarily considered when investigating the pathophysiology of fragility in clinical research beyond the influence of bone mass and geometry.

## Stiffness, Strength and Toughness

These are three important mechanical properties of a material, describing different behaviors. Since there is some confusion in the literature about the exact meaning of these terms, they are (at least qualitatively) defined in Fig. 1.

**Fig. 2:**
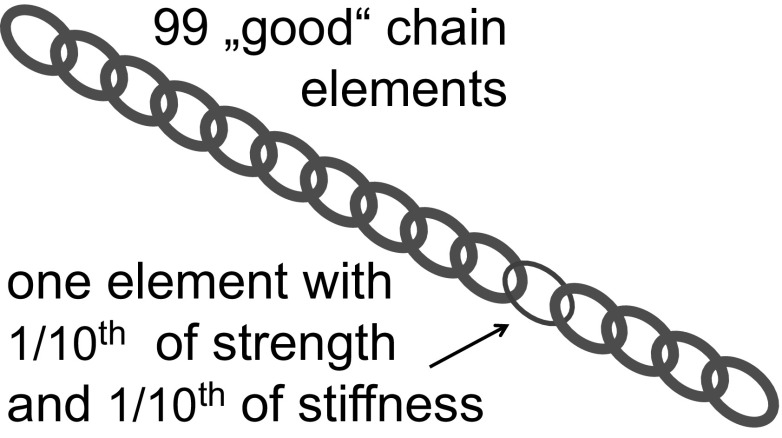
Weakest link problem. As an example, one may consider a chain consisting of 99 “good” elements and 1 with just 1/10th of the stiffness and 1/10th of the strength. The overall stiffness of the chain is given by the inverse average of the stiffness of its elements, that is, it corresponds to 100/(99 + 1/0.1) = 92 % of the stiffness of a good element. The overall strength, however, is completely dominated by the single bad element and therefore drops to 10 % of the strength of good elements. This shows that a small defect affects strength much more than stiffness

While toughness and stiffness are opposing parameters, mineralized bone matrix is a good compromise between these two [8]. Stiffness is needed to prevent bending of bones under the weight of the body and toughness to prevent fractures under minimal impact. Both strength and toughness are properties related to fracture, while stiffness describes the behavior of the material at small loads. In inhomogeneous materials such as mineralized bone matrix, overall stiffness is an average of the local values of the stiffness. Depending on the structure, this average is not always linear but more complex, so that numerical techniques are needed to calculate the average stiffness based on the properties of the constituents and their distribution in space [9, 10]. Nevertheless, the average stiffness is always between the lowest and the highest value in the material, more precisely between two boundaries sometimes called the Voigt and Reuss limits [11, 12]. Unfortunately, the situation is much more complex when it comes to strength and toughness. Indeed, the quantities cannot be calculated just based on the corresponding values of the constituents, but depend on the nucleation and progression of cracks. These are governed by tiny defects in the materials, such as pores, preexisting microdamage, interfaces, fiber directions and so on. Hence, strength and toughness are less described by the properties of the constituents than by their interfaces. The best analogy to rationalize this difficulty is the well-known problem of the weakest link in the chain (Fig. 2).

This limits the possibilities of predicting the strength of a material. In brittle ceramics, for example, strength is limited by the size of the largest pore in the specimen, so that strength in ceramic parts is really a statistical quantity, as it depends on the chance of finding a large defect [13, 14]. Another interesting observation in ceramics is that strength becomes dependent on specimen size at the nanoscale. Indeed, smaller specimens will have smaller defects and therefore a higher strength [15]. This is quite relevant for bone matrix, where the size of mineral particles is so small that they are expected to have the highest possible strength (the intrinsic strength governed by molecular bonds only) of carbonated apatite [16].

The situation gets even more complicated when discussing the origin of toughness. Toughness actually describes the energy dissipated when a crack runs through a material, and this energy should be as large as possible. Ideally, if the capability of the material to dissipate energy is larger than the energy of the impact, a crack will either not be nucleated or dissipate sufficient energy during propagation so that it eventually stops before the bone is broken. Incomplete fractures leading to material damage only can be tolerated by bone because of its remodeling capacity. Indeed, the defects remaining from incomplete fractures will eventually be repaired by this process [17].

Due to these complexities, no complete theory exists that would allow predicting the fracture properties of an extremely inhomogeneous material, such as the mineralized bone matrix. Indeed, inhomogeneities may initiate unwanted cracks, but they may also be favorable, as they can hinder the propagation of cracks, thus dissipating energy and stopping or slowing the crack. Therefore, it is not obvious a priori whether the inhomogeneity of bone material is an advantage or a drawback, but recent research demonstrates that its complex material architecture is actually beneficial for preventing fractures [18–20]. The most important point, however, is that stiffness can be considered an average property of the inhomogeneous material, but strength and, to an even greater extent, toughness cannot. These are largely controlled by interface properties and inhomogeneities. Because those can be very localized in the material, potential therapies acting on these hot spots (schematized by the red chain element in Fig. 2) may have a large effect on fragility, even without an important increase of bone mass.

## Multiscale Interfaces in Bone Material

The multiscale structure of bone has been reviewed multiple times [21–24]. The purpose of this section is to briefly introduce the basic building blocks, different bone types and various interfaces in compact bone material that may be relevant for its toughening.

On the nanometer scale, the mineralized bone matrix is a composite material consisting of an inorganic and organic phase. The inorganic phase contributes about 50–74 % to the total weight [25]. The organic phase represents about 30 wt% and consists mainly of collagen type I, but also of different non-collagenous proteins, proteoglycans and lipids. The remainder, about 8–10 wt%, is water.

**Fig. 3:**
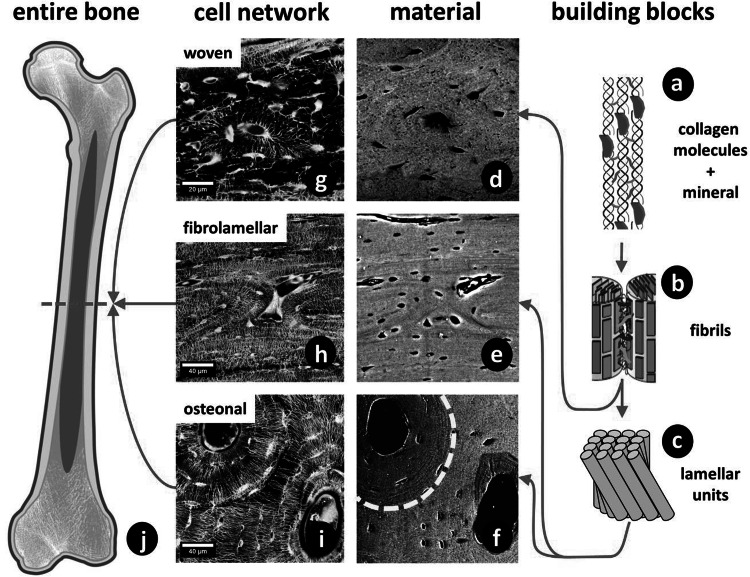
Hierarchical structure of bone. **a** Collagen molecules, connected by crosslinks, and embedded mineral particles. **b** Collagen fibrils connected by an extrafibrillar matrix rich in proteoglycans and non-collagenous proteins. **c** Lamellar units form (**f**) osteonal and (**e**) fibrolamellar bone. The *dashed line* in **f** indicates a cement line around the osteon. **d** Woven bone lacks such highly organized fibril arrangement. **g**–**i** Corresponding cell networks to images **d**–**f**. All images show sections of long bones (**j**) in different species (woven: murine; fibrolamellar: ovine; osteonal: equine). Different bone types and different size levels exhibit varying interfaces. The *red arrows* indicate the sequence of hierarchical levels (from the smallest to largest) for the different bone types (Color figure online)

The collagen molecules are staggered axially by a periodic distance of about 64–67 nm and are made up of three polypeptide chains that form a triple helix with a thickness of 1.5 nm and length of 300 nm (Fig. 3a) [26, 27]. The staggering of molecules leads to overlap zones and gap zones, exhibiting a banded structure visible in transmission electron microscopy. At the interface between collagen molecules there are crosslinks, mainly connecting the telopeptide ends of the triple-helical molecules to one or two neighboring molecules [28]. These enzymatic and non-enzymatic crosslinks stabilize the structure and mechanically reinforce the collagen. These crosslinks vary with tissue type and maturation and also with certain diseases [29].

Within the collagen fibrils, the mineral is embedded in the form of thin hydroxyapatite platelets, whose thickness ranges from 2 to 7 nm, length from 15 to 200 nm and width from 10 to 80 nm [25]. Their long axis is mainly parallel to the long axis of the collagen fibrils. Hydroxyapatite in mineralized tissues is impure and deficient in calcium but enriched in carbonate replacing phosphate ions at several lattice sites. Besides carbonate, magnesium together with other elements can be present in hydroxyapatite. At the interface between the mineral and collagen, ionic bonds connect side-chain carboxyls of the protein and calcium ions in the mineral particles [30] (Fig. 3b). Furthermore, the backbone carbonyls of proline residues form complexes with the mineral’s calcium ions [31]. There is also a plate-like extrafibrillar mineral present, coating the collagen fibrils.

At the next higher level of the hierarchy, several collagen molecules form fibrils with diameters of about 100 nm. The interface layer between neighboring fibrils is filled with extrafibrillar matrix and contains noncollagenous proteins such as osteopontin and proteoglycans such as decorin [6]. The mineralized collagen fibril in mineralized tissues is typically 50–200 nm in diameter and represents the basic building block of bone. These mineralized collagen fibrils can be arranged in different ways, forming fiber bundles with varying degrees of organization. Woven bone is characterized by randomly oriented fibril arrays (Fig. 3d). This is typically found in quickly formed bone, for example, in embryonic or young bones and at repair sites after fractures. It is the mechanically weakest bone type and often substituted during remodeling by other more organized bone types [32].

Bone with the highest degree of organization at the fibril organization level is lamellar bone, which is formed more slowly than other bone types. Lamellar bone can be formed by the remodeling of pre-existing (sometimes woven) bone to create secondary osteons [33], in which lamellae are wrapped around central blood vessels (Fig. 3f). Generally, fibrils in lamellar bone are organized according to a rotated plywood structure, with alternating layers of rotating collagen fibril orientations within each lamella [34, 35]. Recently, the degree of alignment was also shown to change between layers [36]. Osteons, as well as most bone packets formed during a remodeling event, are surrounded by cement lines, which are often more highly mineralized [37] than the surrounding matrix and rich in non-collagenous proteins, such as osteopontin [38]. Cement lines have also been reported at the boundaries of primary osteons (e.g., in antler bone) [6]. While compact bone in adult humans is to the greatest extent lamellar, rodent bone is often characterized as predominantly woven with only small layers of lamellar bone on the endo- or periosteal side of the cortex [39, 40].

A bone type intermediate between woven and lamellar is fibrolamellar bone (also called plexiform bone), found in the bovine or ovine skeleton (Fig. 3e) [32, 41]. Individual parallelfibered units of fibrollamellar bone show weak interfaces at the mesoscopic length scale [42]. These interfaces are thought to be relevant to the mechanical and physiological performance of bone and are possibly dominated by a soft organic layer in order to enable physiological processes such as vascularization to occur [42]. In fibrolamellar bone the first formed layer is woven bone, where later a more ordered, lamellar bone matrix is deposited. In some other cases of fast growing bone, such as in the fracture callus, porous woven bone appears first and acts as a substrate for the deposition of lamellar bone [43, 44]. It has been hypothesized that to form the highly organized lamellar matrix, osteoblasts need a scaffold to align and synthesize the extracellular matrix cooperatively [39]. Similar observations were made in antler bone where a woven-bone scaffold grows first and is later filled in by primary osteons [45].

These various bone types are formed by the same type of bone cells, the osteoblasts, which deposit the collagen matrix. Some of the osteoblasts get embedded into the bone matrix and become osteocytes, bone cells that reside in a lacuna-canalicular network during the bone’s lifetime [46]. This network can be visualized using confocal laser scanning microscopy on rhodamine-stained bone samples [40]. Figure 3g–i show the cell network in these different bone types: woven, lamellar, fibrolamellar and osteonal bone.

## Structural Features Controlling Bone Fragility

Bone fragility is primarily governed by the nucleation and propagation of cracks. Hence, to reduce fragility, the material structure should contribute to making it as costly as possible for cracks to form and extend. The more the impact energy is dissipated in the material, the lower the energy available for nucleating and propagating cracks and thus the lower the likelihood the bone will break. Mineral deposition itself does not improve the toughness. Indeed, as visible in the qualitative comparison between tendon and bone (Fig. 1), the deposition of mineral into collagen type I leads to a considerable increase in stiffness (by more than an order of magnitude), but nearly the same strength and a strong decrease in the energy to failure (as estimated by the area under the curve).

Stiffness and toughness are independent mechanical properties (as obvious from Fig. 1). While toughness is most critical, both are required for the stability of bones. Indeed, sufficient stiffness is needed to prevent bone deformities due to bending in some extreme cases of osteomalacia [47]. At the material level, stiffness is governed by the mineral content in the matrix [25, 48], but also by local fiber orientation and even by the size and shape of mineral particles [12, 16]. Moreover, the bone shape and architecture have a strong influence on the stability of the skeleton. The simplest case would be a thinning of the cortex, which weakens long bones in bending. Another example is the trabecular architecture inside a vertebra that is critical to preventing vertebral fractures. Both the cortical thickness and trabecular architecture are macroscopic effects that can be quantified via micro-computed tomography and finite element modeling [49–51]. The variation of stiffness (usually measured by the elastic modulus, see Fig. 1) determines how stresses will distribute throughout the bone material for any given outside load. In ideal cases, the stress would distribute equally; otherwise, failure could occur where the stress is largest. Indeed, there can be situations where stress concentrates in one weak spot of the material (e.g., at pores or cavities), dramatically increasing the probability for crack nucleation and propagation near that spot. This is the reason why the strength of ceramics is limited by intrinsic pores and defects. However, even compact bone is full of cavities and channels, since it houses the osteocyte network, an important endocrine organ in our body [52], as well as the vasculature supporting it. The mechanical effect of cavities in bone has been discussed in detail by Currey and Shahar [53], also referring to earlier literature on this subject. The effect depends largely on their size. For ellipsoidal osteocyte lacunae the stress concentration depends on their orientation with respect to the applied load and, in healthy lamellar bone, the orientation of these ellipsoids is such that no relevant stress concentrations are to be expected [53]. This may change, however, with a pathological (less ordered) bone structure. Blood canals are wider and therefore a larger threat, but special material architectures, such as the lamellar structures surrounding the blood canal in the osteon (see Fig. 3f, i) are probably protecting them from being sources of cracks by reinforcing the perimeter of the canal.

In the following, we concentrate on a discussion of toughening mechanisms encoded in the structure of the bone material, from the molecular to the macroscopic scale. In this context, it is very useful to consider the classification introduced by R.O. Ritchie who distinguishes between intrinsic and extrinsic mechanisms [4, 6]. Intrinsic mechanisms operate at the molecular scale, up to several hundred nanometers. These mechanisms actually retard the nucleation of a crack by dissipating energy through deformation (but not cracking) of the material. This type of mechanism is most prominent in metals, where plastic deformation is at the origin of their fracture resistance [54]. Intrinsic mechanisms slow down not only the nucleation of cracks, but also their progression. The reason is that stress fields, which are largest near the crack tip, are (partially) relaxed by plastic deformation, thus reducing the driving force for crack propagation. Such dissipation mechanisms through plastic and/or viscoelastic deformation also exist in bone material (see below).

**Fig. 4:**
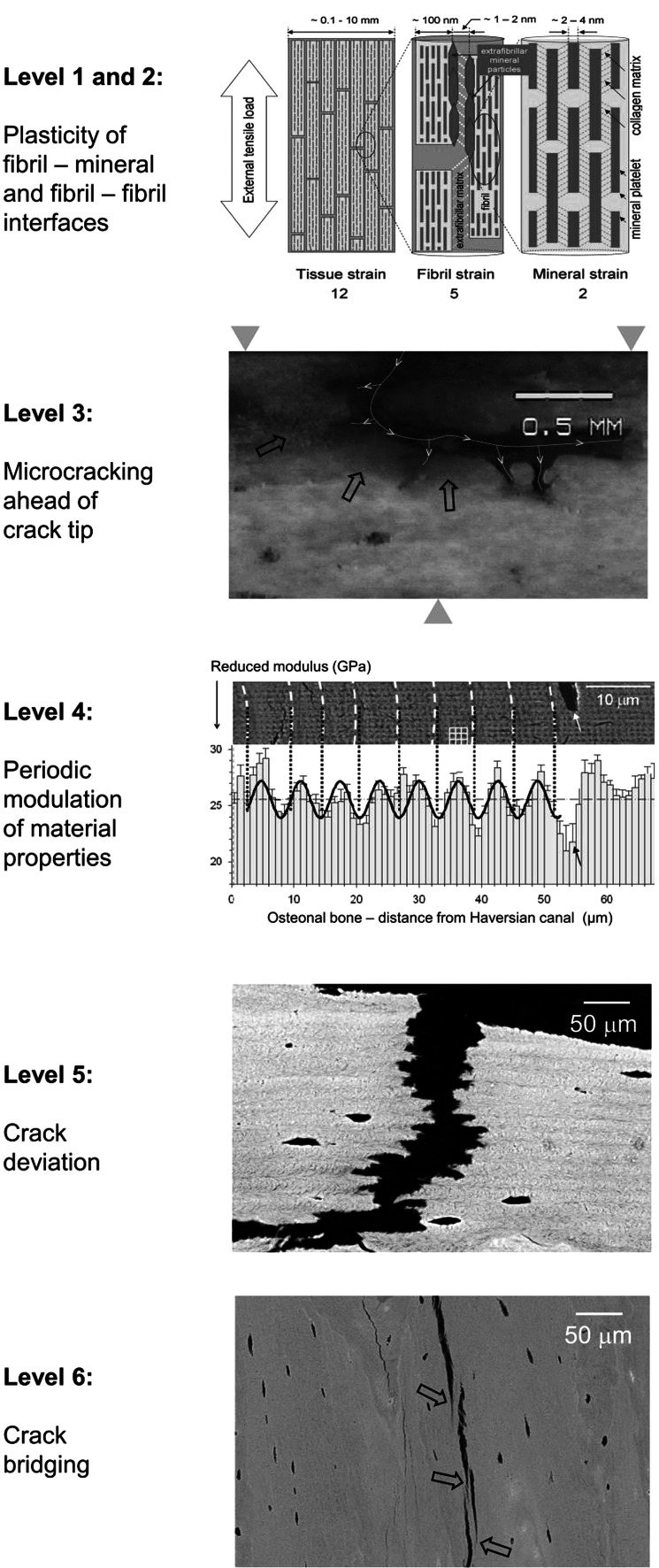
Six well-documented toughening mechanisms operating at different scales [6, 55]. Levels 1 and 2 show the potential of shear deformation between mineral and collagen (few nanometer scale) and between collagen fibrils (100-nm scale). The numbers under the graph indicate the relative magnitude of the stains at the different levels (12:5:2). Level 3 shows penetration of rhodamine stain (*black arrows*) into compartments well ahead of the crack tip, most likely because of the formation of micro-cracks. The *yellow line* indicates the crack that is deviating and splitting. Level 4 shows the periodic modulation of the indentation modulus within successive lamellae in the osteon. Level 5 shows crack undulating deviation across lamellar bone. Level 6 shows crack bridging by uncracked ligaments (*black arrows*). Pictures are adapted from [57] for level 1/2, from [55, 58] for levels 3, 5 and 6 (with permission from Macmillan Publishers, Ltd.), and from [59] for level 4 (with permission from Cambridge University Press)

Extrinsic toughening mechanisms are known from ceramics and composites, which are not capable of plastic deformation and where crack deviation, crack bridging and microcracking are essential mechanisms that slow the propagation of existing cracks. They have been shown to be very important also in bone. Figure 4 summarizes these mechanisms with increasing feature size (based on tables from [55] and [4] ). All these mechanisms have been described previously in some detail. This does not exclude, however, the existence other toughening mechanisms. A study on the scale dependence of toughness has just been published [56].

### Levels 1 and 2: Mineral-Collagen and Fibril-Fibril Interfaces

Mineral particles are essentially plate-like and embedded in and around collagen fibrils. An important feature is the co-alignment of the apatite c-axis (long dimension of the platelets) with the collagen molecule direction. This allows an intimate interaction between the collagen and mineral. In-situ deformation experiments with diffraction of synchrotron radiation have shown that there is shear in the collagen between the mineral particles, as fibrils extend more under tension than the mineral particles embedded in them [57]. Similarly, there is shear between parallel mineralized collagen fibrils as in a tensile test the tissue as a whole extends more than the fibrils of which it is composed (see Fig. 4) [57, 60]. Typically, when the bone strains by 1.2 %, the fibrils strain 0.5 % and the mineral 0.2 % (see Fig. 4, top). The difference in strain is taken up by shear between mineral and collagen [61] or in the matrix between fibrils [60]. This matrix shear breaks non-covalent bonds; thus, it dissipates energy during deformation and contributes to the bone toughness. This breaking of sacrificial bonds has been reported based on various types of analyses from atomic force microscopy [62, 63] to temperature-dependent deformation [64].

It is important to note that this dissipation requires parallel fibrils in a significantly large bone volume to allow side-by-side gliding. This is the case in lamellar or plexiform bone. It is likely that the disordered woven bone structure does not allow for this deformation mode, and, although there is no direct experimental evidence for this, it is likely to be more brittle [65]. It is important to note in this context that murine bone is largely woven, while human bone is lamellar, a difference that could well be reflected in their relative fragility. Alterations in crosslink patterns have also been linked to bone fragility [66]. Moreover, additional (unspecific) crosslinks (such as occur in diabetes [29]) could also hinder the shear deformation of the matrix, thus reducing the toughness of bone at this level.

### Level 3: Microcrack Formation Ahead of Crack Tips

The formation of micro-cracks ahead of crack tips has been reported in many studies [67, 68]. These are on the scale of several hundred nanometers to microns and appear by a separation between fibrils and lamellae. The energy consumed in forming these additional internal interfaces reduces the energy available to drive the crack forward during the impact and thus reduces the crack driving force. Figure 4 (level 3) shows the absorption of rhodamine stain into the micro-cracked area formed ahead of the macroscopic crack tip in the form of a stained halo (arrows in the figure). One idea is that these microcracks are later removed during a remodeling process, which restores the energy dissipation capacity of the material [17]. Indeed, accumulation of microcracks would gradually reduce the toughness of the material and contribute to fatigue fractures. A whitening of bone tissue (related to the scattering of light by defects in the size-range of the light wavelength) has also been reported upon strong deformation of bone [69]. Conceptually, the energy dissipation by microcracks in bone is similar to the crazing described for some polymers [70]. This process is likely to dissipate large amounts of energy, although exact numbers cannot be given. Microdamage has also been reported to stimulate remodeling [71] and thus the repair of damaged material by replacement. This means that exercise can have a beneficial effect by stimulating bone remodeling.

### Level 4: Periodic Variation of Material Properties

Recent work has highlighted the fact that a periodic variation of elastic modulus reduces the crack driving force [72–74]. The rationale is the following: while a downwards gradient of the elastic modulus favors crack propagation, an upwards gradient hinders it. Thus, the crack gets repeatedly trapped in the valleys of the modulus landscape, which reduces the crack driving force. Taking these recent results [73], the relative crack driving force can be expressed in relation to the one in a homogeneous material with the same average modulus:

$$relative\;crack\;driving\;force = \varPsi \left( {E_{ \text{min} } /E_{\text{avg}} } \right) \lambda /\left( {2a} \right),$$

where *Ψ* is a constant close to 1. According to the data shown in Fig. 4, we can estimate the energy dissipation capacity of lamellar bone structures in the following way: the ratio between the minimum and the average value of the modulus $\left( {E_{ \text{min} } /E_{\text{avg}} } \right)$ can be estimated to about 0.95. The wavelength (that is, the lamellar thickness) is $\lambda$ = 6 μm. Finally, the intrinsic crack length may be estimated from [55] as $a \approx E_{\text{avg}} J/\pi \sigma_{f}^{2} \approx 100 \mu m$, where *J* is the crack extension energy and σ_f_ the strength. With these values the relative crack driving force in lamellar bone is 0.029, that is, about 35 times smaller than in a homogeneous material with the same average modulus. This highlights the fact that an inhomogeneity of bone mechanical properties alone (such as variations in the elastic modulus) leads to energy dissipation during crack propagation and thus to increased toughness [20].

### Level 5: Crack Deviation by Lamellae and at Cement Lines

In addition to the effect of modulus variation, there is the possibility of energy dissipation by crack deviation between the lamellae and the formation of microcracks. Several studies [55, 75, 76] have shown that the deviation of cracks at relatively weak interfaces, such as between lamellae or at cement lines, dissipates energy and reduces the crack driving force. This is a well-known effect in composites and makes a significant contribution to toughness. Also interesting in this context is the discussion of whether cement lines have a higher or lower modulus than bone [77, 78]. While stiff (and brittle) interfaces may lead to crack deviation by delamination of the interface, sufficiently soft interfaces are able to slow down or even stop the crack without crack deviation, thus without much additional damage by the above-described modulus variation mechanism.

Figure 4 shows how the crack follows a zig-zag path when crossing a lamellar structure perpendicular to the lamellae. A quantitative evaluation showed that the crack propagation energy is about two orders of magnitude higher when the crack runs perpendicular to the lamellae than when it runs parallel to them [55]. This huge factor probably includes the effect described at level 4. The fact that a concentric arrangement of lamellae surrounds the Haversian canal in an osteon [35, 79] makes the extension of cracks starting from the canal very difficult and thus unlikely. This is probably a very effective way to mechanically shield the blood vessels and capillaries in the bone [33], therefore preventing them from being sources of cracks as they would be in many porous brittle materials [80, 81].

### Level 6: Crack Ligament Bridging

Finally, toughness is also increased by fibers or ligaments bridging the crack in the process zone behind the crack front (see Fig. 4, lowest panel). As seen in this scanning electron microscopic picture, fiber orientation starts to deviate the crack (close to the arrows), but instead of following a zig-zag path as in level 5, a new crack is nucleated ahead of the old one, leaving bridges between the two pieces of material. These bridges take up some of the applied load, reducing the stress at the crack tip and therefore the driving force for crack propagation [82].

## Implications for Diseases Affecting Bone Fragility

Many metabolic or genetic bone diseases exist, which involve increased bone fragility and consequently increased fracture risk, although different pathophysiological mechanisms interfere with bone matrix properties at different hierarchical levels. Bone mass and geometry are, of course, a major determinant of the mechanical behavior of bone. However, at a given bone mass and geometry, fragility is due to a reduced capacity of the bone material to dissipate the energy of an impact through the various mechanisms as described in Fig. 4 and in the preceding section. Therefore, increased fragility may result from an impairment of any of the mechanisms described above, including the interface between mineral and collagen, between fibrils, between lamellae or even at higher scales. While a discussion of the effect of bone diseases on fragility is not the primary goal of this review, we will finish here with several sometimes speculative thoughts about which process might be most affected in various disease patterns.

An additional interesting aspect is the ability of bone to adapt to mechanical stimuli, which results in compensatory reactions. For example, to reduce the increased fracture risk caused by a disturbance in collagen, more bone mass may be formed. This implies that structural defects, which interfere with the biomechanical competence of bone, may trigger compensatory mechanisms through adaptation to mechanical stimuli, which are attenuating these defects. For example, it has been reported in mice that genetic variants affecting one trait may be compensated by coordinated changes in other traits [83]. In osteomalacia, where decreased mineralization results in deformation and sometimes even pseudofractures (also called Looser’s zones), increased volumes of osteoid and poorly mineralized matrix are often found. In advanced stages of “hypovitaminosis D osteopathy,” histomorphometric analysis describes accumulation of unmineralized osteoid as well as a cortical bone deficit combined with signs of secondary hyperparathyroidism, frequently with fibrous tissue in the marrow spaces [84]. At the material level poorly mineralized matrix reduces the average stiffness, and one could speculate that the increase of osteoid synthesis as well as the formation of fibrous tissue in the marrow spaces may be (ineffective) compensatory effects.

In contrast, postmenopausal osteoporosis is characterized by lower bone mass and microarchitectural deterioration. Low bone mass alone, of course, increases fragility even with identical bone material properties. This leads to fractures, primarily in the thoracic and lumbar vertebrae, proximal femur and distal forearms in combination with low energy trauma. So far, it remains unknown whether additional structural defects of matrix properties contribute to fragility in postmenopausal osteoporosis, although recent spectroscopic data have revealed some evidence in favor of this assumption [85–87]. When comparing fracture to non-fracture cases with the same low bone mass in elderly individuals, the mineral-collagen composite was found stiffer in the fracture cases at a given mineral content [88]. This was interpreted as an alteration of the organic matrix in these cases. Indeed, a stiffer (perhaps more strongly crosslinked) organic matrix would make the overall composite material more brittle. In addition, bone remodeling measured in terms of activation frequency in histomorphometry is generally increased in postmenopausal osteoporosis, although to a widely varying extent. This leads to an on average lower mineral content in the bone matrix, which can be corrected by antiresorptive treatments and vitamin D [48, 89, 90]. It is not exactly known how the bone packets with lower mineral content accumulating in postmenopausal osteoporosis influence fragility, but the uneven mineral distribution may result in stress concentration at the edges of these packets, facilitating crack nucleation. Supplementation with calcium and vitamin D increases the mineral content of the bone matrix in postmenopausal osteoporosis without an effect on the bone volume [91], which could potentially have an effect on fragility beyond changes in bone mass.

Other metabolic bone diseases such as hypophosphatemic osteopathy, gastrointestinal bone disease and renal osteodystrophy have an even more complex pathophysiology. Phosphate depletion of any etiology leads to disturbances in mineralization, morphometrically comparable to severe hypovitaminosis D. Detailed analyses of the bone material are mostly lacking for these cases, although mechanical deficits are evident.

Aging has been reported to lead to a modification of the organic matrix and, in particular, to changes in crosslinks [66], which may be at the origin of increased fragility [29, 92]. Indeed, due to the increase of advanced glycation end products (AGEs) crosslinking during aging, the material properties of mature tissue differ from newly synthesized matrix. This phenomenon is clearly linked to an impairment of toughening mechanisms at levels 1 and 2. Changes in collagen structure underlie an age-related reduction in bone toughness, increasing fracture risk independent of bone mineral density [5]. Age-related changes in the balance of bone formation and resorption have been shown to be influenced also by mechanical loading [93], which may influence bone material properties and fragility.

Diabetes has been shown to induce bone fragility [29, 94]. The origin of this effect is not fully elucidated, but it is known that AGEs lead to extra crosslinks, which are likely to block the gliding between fibrils (level 2). Recent data using synchrotron X-ray scattering seem to support this idea [95]. Hence, bone fragility in diabetes could mainly originate from levels 1 and 2, but it is not excluded that higher levels might also be affected.

Fragility has also been observed in fluorotic bone. In this case, the interaction between the mineral and collagen has been altered [48, 96–98], which may be at the origin of fragility, as the bone density is increased by fluoride treatment [99]. It is also possible that the strong anabolic effect of fluoride [100] compensates partially for the detriment due to the impaired material properties.

It is quite a mystery that many types of osteogenesis imperfecta lead to bone fragility and share similar phenotypes, such as low bone mass and increased mineral content, despite a wide variety in genetic mutations. Indeed, a slightly enhanced mineral concentration is a hallmark of these diseases, and a reduced alignment of collagen has been observed in both mouse models and patient biopsies [48, 101–103]. While this minute increase in mineral content (by 1 or 2 %) can hardly account for the decreased toughness of the material, the reduced degree of alignment means that the dissipation according to levels 4 and 5 in Fig. 4 is likely reduced because the lamellar character of the bone is less pronounced. As a matter of fact, woven bone (see Fig. 3), which lacks lamellar structure, is likely to lose one or two orders of magnitude in toughness according to the estimates in the previous section. Hence, the putative inability in the case of OI to remodel primary bone into a high-quality lamellar structure may have a stronger effect on bone fragility than the collagen defect itself. Several recent reports actually point into this direction [65, 102]. A different case is pyknodysostosis, where a reduction of lamellar order in the bone the tissue leads to fragility, despite high bone mass [104].

The discussion in this section was not meant to give any new fundamental insights into any of the mentioned bone diseases. We hope that it demonstrates how the catalog of toughening processes reviewed in this article could potentially be used to carry out a more structured search for the needle in the haystack and uncover the origin of fragility, which may improve the diagnosis and treatment of these conditions.

## References

[1] Forlino A, Cabral WA, Barnes AM, Marini JC (2011). New perspectives on osteogenesis imperfecta. Nat Rev Endocrinol.

[2] Rauch F, Glorieux FH (2004). Osteogenesis imperfecta. Lancet.

[3] Sambrook P, Cooper C (2006). Osteoporosis. Lancet.

[4] Ritchie RO (2011). The conflicts between strength and toughness. Nat Mater.

[5] Burr D (2002). The contribution of the organic matrix to bone’s material properties. Bone.

[6] Launey ME, Buehler MJ, Ritchie RO (2010). On the mechanistic origins of toughness in bone. Annu Rev Mater Res.

[7] Fratzl P (2008). Collagen : structure and mechanics.

[8] Wegst U, Ashby M (2004). The mechanical efficiency of natural materials. Phil Mag.

[9] MacNeil JA, Boyd SK (2008). Bone strength at the distal radius can be estimated from high-resolution peripheral quantitative computed tomography and the finite element method. Bone.

[10] Van Rietbergen B, Weinans H, Huiskes R, Odgaard A (1995). A new method to determine trabecular bone elastic properties and loading using micromechanical finite-element models. J Biomech.

[11] Bendsoe MP, Sigmund O (1999). Material interpolation schemes in topology optimization. Arch Appl Mech.

[12] Jager I, Fratzl P (2000). Mineralized collagen fibrils: a mechanical model with a staggered arrangement of mineral particles. Biophys J.

[13] Danzer R, Supancic P, Pascual J, Lube T (2007). Fracture statistics of ceramics–Weibull statistics and deviations from Weibull statistics. Eng Fract Mech.

[14] Khandaker M, Ekwaro-Osire S (2013). Weibull Analysis of Fracture Test Data on Bovine Cortical Bone: Influence of Orientation. Int J Biomater.

[15] Davidge RW, Evans AG (1970). The strength of ceramics. Mater Sci Eng.

[16] Gao HJ, Ji BH, Jager IL, Arzt E, Fratzl P (2003). Materials become insensitive to flaws at nanoscale: lessons from nature. P Natl Acad Sci USA.

[17] Taylor D, Hazenberg JG, Lee TC (2007). Living with cracks: damage and repair in human bone. Nat Mater.

[18] Fratzl P (2008). Bone fracture - when the cracks begin to show. Nat Mater.

[19] Ritchie RO (2010). How does human bone resist fracture?. Ann Ny Acad Sci.

[20] Tai K, Dao M, Suresh S, Palazoglu A, Ortiz C (2007). Nanoscale heterogeneity promotes energy dissipation in bone. Nat Mater.

[21] Fratzl P, Weinkamer R (2007). Nature’s hierarchical materials. Prog Mater Sci.

[22] Meyers MA, Chen PY, Lin AYM, Seki Y (2008). Biological materials: structure and mechanical properties. Prog Mater Sci.

[23] Rho J-Y, Kuhn-Spearing L, Zioupos P (1998). Mechanical properties and the hierarchical structure of bone. Med Eng Phys.

[24] Weiner S, Wagner HD (1998). The material bone: structure mechanical function relations. Annu Rev Mater Sci.

[25] Fratzl P, Gupta HS, Paschalis EP, Roschger P (2004). Structure and mechanical quality of the collagen-mineral nano-composite in bone. J Mater Chem.

[26] Hulmes DJS, Wess TJ, Prockop DJ, Fratzl P (1995). Radial packing, order, and disorder in collagen fibrils. Biophys J.

[27] Petruska JA, Hodge AJ (1964). Subunit model for tropocollagen macromolecule. P Natl Acad Sci USA.

[28] Avery NC, Bailey AJ, Fratzl P (2008). Restraining cross-links responsible for the mechnical properties of collagen fibers: natural and artificial. Collagen - structure and mechanics.

[29] Saito M, Marumo K (2010). Collagen cross-links as a determinant of bone quality: a possible explanation for bone fragility in aging, osteoporosis, and diabetes mellitus. Osteoporos Int.

[30] Beniash E (2011). Biominerals-hierarchical nanocomposites: the example of bone. Wires Nanomed Nanobiotechnol.

[31] Elangovan S, Margolis HC, Oppenheim FG, Beniash E (2007). Conformational changes in salivary proline-rich protein 1 upon adsorption to calcium phosphate crystals. Langmuir.

[32] Currey J (2003). The many adaptations of bone. J Biomech.

[33] Currey JD (2002) Bones: structure and mechanics. Princeton University Press

[34] Portigliatti Barbos M, Bianco P, Ascenzi A, Boyde A (1984). Collagen orientation in compact bone: II. Distribution of lamellae in the whole of the human femoral shaft with reference to its mechanical properties. Metab Bone Dis Relat Res.

[35] Weiner S, Traub W, Wagner HD (1999). Lamellar bone: structure–function relations. J Struct Biol.

[36] Reznikov N, Shahar R, Weiner S (2014). Bone hierarchical structure in three dimensions. Acta Biomater.

[37] Skedros JG, Holmes JL, Vajda EG, Bloebaum RD (2005). Cement lines of secondary osteons in human bone are not mineral-deficient: new data in a historical perspective. Anat Rec A Discov Mol Cell Evol Biol.

[38] Sodek J, Ganss B, McKee M (2000). Osteopontin. Crit Rev Oral Biol Med.

[39] Kerschnitzki M, Wagermaier W, Liu YF, Roschger P, Duda GN, Fratzl P (2011). Poorly ordered bone as an endogenous scaffold for the deposition of highly oriented lamellar tissue in rapidly growing ovine bone. Cells Tissues Organs.

[40] Kerschnitzki M, Wagermaier W, Roschger P, Seto J, Shahar R, Duda GN, Mundlos S, Fratzl P (2011). The organization of the osteocyte network mirrors the extracellular matrix orientation in bone. J Struct Biol.

[41] Francillon Vieillot H, De Buffrénil V, Castanet Jd, Géraudie J, Meunier F, Sire J, Zylberberg L, De Ricqlès A (1990) Microstructure and mineralization of vertebrate skeletal tissues. In: Carter JG (ed) Skeletal biomineralization: patterns, processes and evolutionary trends. Van Nostrand Reinhold, New York, pp 471–530

[42] Seto J, Gupta HS, Zaslansky P, Wagner HD, Fratzl P (2008). Tough lessons from bone: extreme mechanical anisotropy at the mesoscale. Adv Funct Mater.

[43] Hoerth RM, Seidt BM, Shah M, Schwarz C, Willie BM, Duda GN, Fratzl P, Wagermaier W (2014). Mechanical and structural properties of bone in non-critical and critical healing in rat. Acta Biomater.

[44] Liu Y, Manjubala I, Schell H, Epari DR, Roschger P, Duda GN, Fratzl P (2010). Size and habit of mineral particles in bone and mineralized callus during bone healing in sheep. J Bone Miner Res.

[45] Krauss S, Wagermaier W, Estevez JA, Currey JD, Fratzl P (2011). Tubular frameworks guiding orderly bone formation in the antler of the red deer (*Cervus elaphus*). J Struct Biol.

[46] Bonewald LF (2011). The amazing osteocyte. J Bone Miner Res.

[47] Holick MF (2006). Resurrection of vitamin D deficiency and rickets. J Clin Invest.

[48] Roschger P, Paschalis EP, Fratzl P, Klaushofer K (2008). Bone mineralization density distribution in health and disease. Bone.

[49] Prendergast P (1997). Finite element models in tissue mechanics and orthopaedic implant design. Clin Biomech.

[50] Stauber M, Nazarian A, Müller R (2014). Limitations of global morphometry in predicting trabecular bone failure. J Bone Miner Res.

[51] Zysset PK (2003). A review of morphology–elasticity relationships in human trabecular bone: theories and experiments. J Biomech.

[52] DiGirolamo DJ, Clemens TL, Kousteni S (2012). The skeleton as an endocrine organ. Nat Rev Rheumatol.

[53] Currey JD, Shahar R (2013). Cavities in the compact bone in tetrapods and fish and their effect on mechanical properties. J Struct Biol.

[54] Kumar K, Van Swygenhoven H, Suresh S (2003). Mechanical behavior of nanocrystalline metals and alloys. Acta Mater.

[55] Peterlik H, Roschger P, Klaushofer K, Fratzl P (2006). From brittle to ductile fracture of bone. Nat Mater.

[56] Zimmermann EA, Gludovatz B, Schaible E, Busse B, Ritchie RO (2014). Fracture resistance of human cortical bone across multiple length-scales at physiological strain rates. Biomaterials.

[57] Gupta HS, Seto J, Wagermaier W, Zaslansky P, Boesecke P, Fratzl P (2006). Cooperative deformation of mineral and collagen in bone at the nanoscale. Proc Natl Acad Sci.

[58] Peterlik H, Roschger P, Klaushofer K, Fratzl P (2006). Orientation dependent fracture toughness of lamellar bone. Int J Fract.

[59] Gupta H, Stachewicz U, Wagermaier W, Roschger P, Wagner H, Fratzl P (2006). Mechanical modulation at the lamellar level in osteonal bone. J Mater Res.

[60] Gupta HS, Wagermaier W, Zickler GA, Raz-Ben Aroush D, Funari SS, Roschger P, Wagner HD, Fratzl P (2005). Nanoscale deformation mechanisms in bone. Nano Lett.

[61] Gupta H, Krauss S, Kerschnitzki M, Karunaratne A, Dunlop J, Barber A, Boesecke P, Funari S, Fratzl P (2013). Intrafibrillar plasticity through mineral/collagen sliding is the dominant mechanism for the extreme toughness of antler bone. J Mech Behav Biomed Mater.

[62] Fantner GE, Hassenkam T, Kindt JH, Weaver JC, Birkedal H, Pechenik L, Cutroni JA, Cidade GA, Stucky GD, Morse DE (2005). Sacrificial bonds and hidden length dissipate energy as mineralized fibrils separate during bone fracture. Nat Mater.

[63] Thurner PJ, Katsamenis OL (2014). The role of nanoscale toughening mechanisms in osteoporosis. Curr Osteoporos Rep.

[64] Gupta HS, Fratzl P, Kerschnitzki M, Benecke G, Wagermaier W, Kirchner HO (2007). Evidence for an elementary process in bone plasticity with an activation enthalpy of 1 eV. J R Soc Interface.

[65] Carriero A, Zimmermann EA, Paluszny A, Tang SY, Bale H, Busse B, Alliston T, Kazakia G, Ritchie RO, Shefelbine SJ (2014). How tough is brittle bone? Investigating osteogenesis imperfecta in mouse bone. J Bone Miner Res.

[66] Paschalis EP, Shane E, Lyritis G, Skarantavos G, Mendelsohn R, Boskey AL (2004). Bone Fragility and Collagen Cross-Links. J Bone Miner Res.

[67] Gupta H, Zioupos P (2008). Fracture of bone tissue: the ‘hows’ and the ‘whys’. Med Eng Phys.

[68] Zioupos P, Currey J (1998). Changes in the stiffness, strength, and toughness of human cortical bone with age. Bone.

[69] Thurner PJ, Erickson B, Jungmann R, Schriock Z, Weaver JC, Fantner GE, Schitter G, Morse DE, Hansma PK (2007). High-speed photography of compressed human trabecular bone correlates whitening to microscopic damage. Eng Fract Mech.

[70] Friedrich K (1983). Crazes and shear bands in semi-crystalline thermoplastics. In: Crazing in Polymers.

[71] Burr D (2011). Why bones bend but don’t break. J Musculoskelet Neuronal Interact.

[72] Fratzl P, Gupta HS, Fischer FD, Kolednik O (2007). Hindered crack propagation in materials with periodically varying young’s modulus—lessons from biological materials. Adv Mater.

[73] Kolednik O, Predan J, Fischer F, Fratzl P (2014). Improvements of strength and fracture resistance by spatial material property variations. Acta Mater.

[74] Kolednik O, Predan J, Fischer FD, Fratzl P (2011). Bioinspired Design Criteria for Damage-Resistant Materials with Periodically Varying Microstructure. Adv Funct Mater.

[75] Koester KJ, Ager JW, Ritchie RO (2008). The true toughness of human cortical bone measured with realistically short cracks. Nat Mater.

[76] Nalla R, Kruzic J, Kinney J, Balooch M, Ager Iii J, Ritchie R (2006). Role of microstructure in the aging-related deterioration of the toughness of human cortical bone. Mater Sci Eng C.

[77] Skedros JG, Holmes JL, Vajda EG, Bloebaum RD (2005). Cement lines of secondary osteons in human bone are not mineral-deficient: new data in a historical perspective. Anat Rec A.

[78] Burr DB, Schaffler MB, Frederickson RG (1988). Composition of the cement line and its possible mechanical role as a local interface inhuman compact-bone. J Biomech.

[79] Wagermaier W, Gupta H, Gourrier A, Burghammer M, Roschger P, Fratzl P (2006). Spiral twisting of fiber orientation inside bone lamellae. Biointerphases.

[80] Ovid’Ko I (2007). Review on the fracture processes in nanocrystalline materials. J Mater Sci.

[81] Vandeperre L, Wang J, Clegg† W (2004). Effects of porosity on the measured fracture energy of brittle materials. Phil Mag.

[82] Nalla RK, Kinney JH, Ritchie RO (2003). Mechanistic fracture criteria for the failure of human cortical bone. Nat Mater.

[83] Smith LM, Bigelow EMR, Nolan BT, Faillace ME, Nadeau JH, Jepsen KJ (2014). Genetic perturbations that impair functional trait interactions lead to reduced bone strength and increased fragility in mice. Bone.

[84] Parfitt AM, Qiu SJ, Rao DS (2004). The mineralization index - A new approach to the histomorphometric appraisal of osteomalacia. Bone.

[85] Bailey A, Wotton S, Sims T, Thompson P (1992). Post-translational modifications in the collagen of human osteoporotic femoral head. Biochem Biophys Res Commun.

[86] Malluche HH, Porter DS, Mawad H, Monier-Faugere M-C, Pienkowski D (2013). Low-energy fractures without low T-scores characteristic of osteoporosisa possible bone matrix disorder. J Bone Joint Surg.

[87] Misof BM, Gamsjaeger S, Cohen A, Hofstetter B, Roschger P, Stein E, Nickolas TL, Rogers HF, Dempster D, Zhou H, Recker R, Lappe J, McMahon D, Paschalis EP, Fratzl P, Shane E, Klaushofer K (2012). Bone material properties in premenopausal women with idiopathic osteoporosis. J Bone Miner Res.

[88] Fratzl-Zelman N, Roschger P, Gourrier A, Weber M, Misof B, Loveridge N, Reeve J, Klaushofer K, Fratzl P (2009). Combination of nanoindentation and quantitative backscattered electron imaging revealed altered bone material properties associated with femoral neck fragility. Calcif Tissue Int.

[89] Roschger P, Rinnerthaler S, Yates J, Rodan GA, Fratzl P, Klaushofer K (2001). Alendronate increases degree and uniformity of mineralization in cancellous bone and decreases the porosity in cortical bone of osteoporotic women. Bone.

[90] Zoehrer R, Roschger P, Paschalis EP, Hofstaetter JG, Durchschlag E, Fratzl P, Phipps R, Klaushofer K (2006). Effects of 3-and 5-year treatment with risedronate on bone mineralization density distribution in triple biopsies of the iliac crest in postmenopausal women. J Bone Miner Res.

[91] Fratzl P, Roschger P, Fratzl-Zelman N, Paschalis EP, Phipps R, Klaushofer K (2007). Evidence that treatment with risedronate in women with postmenopausal osteoporosis affects bone mineralization and bone volume. Calcif Tissue Int.

[92] Zioupos P (2001). Ageing human bone: factors affecting its biomechanical properties and the role of collagen. J Biomater Appl.

[93] Birkhold AI, Razi H, Duda GN, Weinkamer R, Checa S, Willie BM (2014). The influence of age on adaptive bone formation and bone resorption. Biomaterials.

[94] Schwartz AV, Sellmeyer DE (2007). Diabetes, fracture, and bone fragility. Curr osteoporos rep.

[95] Fessel G, Li Y, Diederich V, Guizar-Sicairos M, Schneider P, Sell DR, Monnier VM, Snedeker JG (2014). Advanced glycation end-products reduce collagen molecular sliding to affect collagen fibril damage mechanisms but not stiffness. PLoS ONE.

[96] Fratzl P, Roschger P, Eschberger J, Abendroth B, Klaushofer K (1994). Abnormal bone mineralization after fluoride treatment in osteoporosis: a small-angle x-ray-scattering study. J Bone Miner Res.

[97] Fratzl P, Schreiber S, Roschger P, Lafage MH, Rodan G, Klaushofer K (1996). Effects of sodium fluoride and alendronate on the bone mineral in minipigs: a small-angle X-ray scattering and backscattered electron imaging study. J Bone Miner Res.

[98] Gourrier A, Li C, Siegel S, Paris O, Roschger P, Klaushofer K, Fratzl P (2010). Scanning small-angle X-ray scattering analysis of the size and organization of the mineral nanoparticles in fluorotic bone using a stack of cards model. J Appl Crystallogr.

[99] Vestergaard P, Jorgensen N, Schwarz P, Mosekilde L (2008). Effects of treatment with fluoride on bone mineral density and fracture risk-a meta-analysis. Osteoporos Int.

[100] Everett E (2011). Fluoride’s effects on the formation of teeth and bones, and the influence of genetics. J Dent Res.

[101] Fratzl P, Paris O, Klaushofer K, Landis W (1996). Bone mineralization in an osteogenesis imperfecta mouse model studied by small-angle x-ray scattering. J Clin Invest.

[102] Fratzl-Zelman N, Schmidt I, Roschger P, Glorieux FH, Klaushofer K, Fratzl P, Rauch F, Wagermaier W (2014). Mineral particle size in children with osteogenesis imperfecta type I is not increased independently of specific collagen mutations. Bone.

[103] Roschger P, Fratzl-Zelman N, Misof BM, Glorieux FH, Klaushofer K, Rauch F (2008). Evidence that abnormal high bone mineralization in growing children with osteogenesis imperfecta is not associated with specific collagen mutations. Calcif Tissue Int.

[104] Fratzl-Zelman N, Valenta A, Roschger P, Nader A, Gelb BD, Fratzl P, Klaushofer K (2004). Decreased bone turnover and deterioration of bone structure in two cases of pycnodysostosis. J Clin Endocrinol Metab.

